# Enhancing Canola Oil Oxidative Stability Through Nanoencapsulation of *Eryngium planum* Extract in Basil Seed Gum: Effect of Wall Concentration and Core‐to‐Wall Ratio

**DOI:** 10.1002/fsn3.72066

**Published:** 2026-07-17

**Authors:** Nastaran Fallah, Reza Esmaeilzadeh Kenari, Reza Farahmandfar

**Affiliations:** ^1^ Department of Food Science and Technology Sari Agricultural Sciences and Natural Resources University Sari Iran

**Keywords:** antioxidant, basil seed gum, nanoencapsulation, oxidation, release

## Abstract

The growing consumer demand for natural alternatives to synthetic antioxidants has intensified research into plant‐based extracts and encapsulation. This study explores, for the first time, the combined use of *Eryngium planum* extract (EPE) and basil seed gum (BSG) for stabilizing canola oil, focusing on the underexplored factors of wall concentration and core‐to‐wall ratio. The antioxidant activity of the extract was evaluated using DPPH radical scavenging and β‐carotene–linoleic acid bleaching assays. Antioxidant activity increased in a concentration‐dependent manner and reached a level comparable to TBHQ at 2000 ppm. Nanocapsules were prepared using BSG at 1% and 2% concentrations and core‐to‐wall ratios of 1:10 and 1:20. Particle size, polydispersity index (PDI), zeta potential, encapsulation efficiency, and release rate were measured. The smallest particle size (59.8 ± 0.79 nm) and lowest PDI (0.204 ± 0.005) were observed in formulation with 2% BSG and 1:20 ratio, which also showed the highest zeta potential (−36 ± 0.57 mV) and encapsulation efficiency (86.95% ± 0.33%). The highest release rate occurred in the formulation with 1% BSG and 1:10 ratio (BSG1 1:10). Wall concentration exerted a stronger influence on nanocapsule characteristics than the core‐to‐wall ratio. Oxidative stability testing of canola oil (peroxide and p‐anisidine values) indicated that nanocapsules containing the extract were more effective in inhibiting lipid oxidation than the free extract. The BSG1 1:10 sample demonstrated the highest antioxidant performance. Overall, these findings highlight that EPE nanoencapsulation in BSG at 1% concentration with 1:10 ratio offers a promising natural strategy for enhancing canola oil oxidative stability.

## Graphical abstract

The steps of the experiment, different treatment conditions, and the final result are shown in this image.

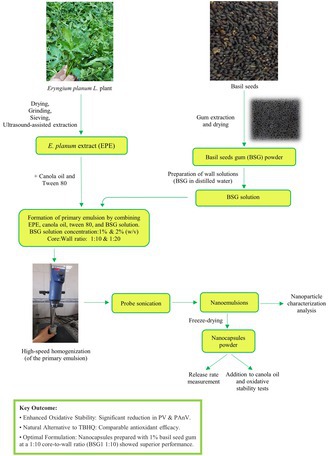

## 1 Introduction

Canola oil is one of the most widely consumed vegetable oils globally, valued for its well‐balanced nutritional profile. Its fatty acid composition is particularly rich in oleic acid (51%), linoleic acid (25%), and alpha‐linolenic acid (14%), all of which contribute to cardiovascular health (Lim et al. 2013). Beyond its lipid profile, canola oil contains significant levels of bioactive compounds such as phytosterols, carotenoids, and tocopherols, which help lower serum LDL levels (Flakelar et al. 2022). Additionally, phenolic compounds like caffeic acid and its derivatives provide further antioxidant and antimutagenic benefits (Mehmood 2015).

Despite these beneficial components, oxidation remains a major chemical degradation process in canola oil during storage. It leads to a decline in nutritional quality and safety and is influenced by factors such as temperature, light exposure, storage duration, and oxygen contact (Ahmadian et al. 2024; Aziminezhad et al. 2024; Erickson et al. 2023; Fadda et al. 2022; Li et al. 2023). To mitigate oxidation, synthetic antioxidants such as butylated hydroxyanisole (BHA), butylated hydroxytoluene (BHT), and tert‐butyl hydroquinone (TBHQ) have been traditionally used due to their high efficacy (Ansorena et al. 2023; Tavakoli et al. 2013). However, growing safety concerns and consumer preference for natural ingredients have driven the search for plant‐based alternatives (Lourenço et al. 2019; Michel et al. 2025).

In this context, *Eryngium planum* L., a perennial herb belonging to the Apiaceae family, has gained attention as a promising candidate. Distributed across various regions including Iran, it has been traditionally used as an edible vegetable, flavoring agent, and natural remedy in different cultures (Kikowska et al. 2012; Paun et al. 2019). Its diverse pharmacological properties, including diuretic, antidiabetic, anti‐inflammatory, and antimicrobial activities, are attributed to its rich profile of bioactive compounds (Arykbayeva et al. 2023). Phytochemical analyses have revealed that this species contains valuable constituents such as triterpenoid saponins, flavonoids (including kaempferol and quercetin glycosides), and phenolic acids (particularly rosmarinic and chlorogenic acids) (Shabani et al. 2022). The notable presence of these antioxidant compounds suggests its potential as a natural preservative for enhancing the oxidative stability of lipid‐based food systems (Kikowska et al. 2012).

To efficiently extract these sensitive bioactives, modern techniques like ultrasound‐assisted extraction (UAE) are employed. UAE offers advantages such as shorter extraction times, reduced solvent consumption, and higher yields compared to conventional methods (Soria and Villamiel 2010). However, even with efficient extraction, the direct incorporation of plant extracts into food products faces challenges, including rapid degradation, interaction with food components, and potential negative effects on sensory quality (Díaz‐Montes 2023).

To address these challenges, nanoencapsulation has emerged as an effective strategy for protecting, stabilizing, and controlling the release of bioactives. This approach entraps active molecules within nanoscale carriers, thereby enhancing stability and bioavailability. Among the available techniques, emulsification is favored for its simplicity and ability to maintain the structural integrity of encapsulated compounds (Jain et al. 2022; Nedovic et al. 2011). Recent studies have successfully utilized plant‐based gums to deliver natural antioxidants into edible oils, demonstrating significantly improved oxidative stability (Aboutalebzadeh et al. 2022; Kenari et al. 2020).

The performance of nanoencapsulation systems is highly dependent on formulation parameters, particularly the type and concentration of the wall material and the core‐to‐wall ratio. These factors critically influence encapsulation efficiency, release kinetics, and the ultimate functional performance of the encapsulated active compound (Vijeth et al. 2019). Among natural polymers, plant‐derived gums are especially attractive as wall materials due to their biocompatibility, emulsifying capacity, and cost‐effectiveness (Wandrey et al. 2010).

In particular, basil seed gum (BSG), derived from the hydrated mucilage of *Ocimum basilicum* L. seeds, has emerged as a promising encapsulation material. The seed coat contains polysaccharides that swell upon hydration to form a gel‐like matrix with excellent stabilizing and film‐forming properties. These characteristics are crucial for forming a protective barrier around bioactive compounds, making BSG particularly suitable for nanoemulsion‐based delivery systems (Guan et al. 2023; Pirsa and Hafezi 2023).

Despite the promising properties of BSG, the influence of its concentration and core‐to‐wall ratio on the encapsulation efficiency, release kinetics, and antioxidant efficacy of *E. planum* extract in canola oil remains unexamined. Filling this knowledge gap is essential for designing controlled‐release systems that effectively mitigate lipid oxidation.

Therefore, this study systematically evaluated how these parameters govern these critical performance metrics. The specific objectives were to: (i) extract and characterize *E. planum* bioactives using ultrasound‐assisted extraction; (ii) evaluate the extract's in vitro antioxidant activity; (iii) fabricate and characterize nanocapsules using BSG at 1% and 2% concentrations with 1:10 and 1:20 core‐to‐wall ratios; and (iv) assess their effectiveness in enhancing canola oil oxidative stability under accelerated storage. The outcomes provide a foundation for designing natural antioxidant delivery systems in the edible oil industry.

## 2 Materials and Methods

### 2.1 Materials

Fresh aerial parts of *Eryngium planum* L. were purchased from a local herbal market in Amol County, Mazandaran Province, Iran. The botanical identity of the plant material was confirmed by a specialist botanist at the Medicinal Plants Research Center of Sari Agricultural Sciences and Natural Resources University. Seeds of *Ocimum basilicum* L. were purchased from the same source. Refined canola oil, free from synthetic antioxidants, was supplied by Ghoncheh Mazandaran Co. (Iran). All analytical‐grade chemicals and reagents used in this study were obtained from Merck (Darmstadt, Germany).

### 2.2 Methods

#### 2.2.1 Extraction of Bioactive Compounds From *E. planum*

Fresh leaves and stems were washed and shade‐dried at ambient temperature (25°C) for 7 days. The dried material was pulverized with a mechanical grinder (Model MX‐AC400, Panasonic, Japan) and sieved to obtain a fine powder. For extraction, 25 g of powder was combined with 250 mL of 80% ethanol (solid‐to‐solvent ratio of 1:10) and subjected to ultrasound‐assisted extraction using a bath sonicator (Soner206, Roker, Taiwan) for 15 min at 37 kHz and 40°C. After centrifugation for 30 min at 7800 rpm (MC50, Behsan, Iran), the supernatant was filtered through Whatman No. 1 paper. The filtrate was concentrated with a rotary evaporator (Model R‐300 R100, Buchi, Switzerland) at 40°C and 200 rpm under vacuum (Alupului and Lavric 2008). All subsequent measurements and formulations were performed in technical triplicate using a single extract batch to ensure consistency.

#### 2.2.2 Phenolic and Flavonoid Content of the Extract

Total phenolic content (TPC) was measured using the Folin–Ciocalteu assay and expressed as mg gallic acid equivalents per gram of sample (Zahed et al. 2023). Briefly, 1 mL of extract was reacted with 5 mL of diluted Folin–Ciocalteu reagent, followed by the addition of 4 mL of 7.5% (w/v) sodium carbonate. The mixture was incubated at 27°C for 30 min, and absorbance was recorded at 760 nm using a UV–Vis spectrophotometer. Total flavonoid content (TFC) was measured using the aluminum chloride colorimetric technique and expressed as mg quercetin equivalents (QE) per gram of extract (Chen, Lin, et al. 2020). Briefly, 1 mL of the extract was mixed with 150 μL of 5% (w/v) sodium nitrite and incubated for 5 min. Then, 300 μL of 10% (w/v) aluminum chloride was added and the mixture was allowed to react for another 5 min. Subsequently, 1 mL of 1 M sodium hydroxide was added, followed by dilution with 1.05 mL of distilled water. The solution was vortexed briefly, and absorbance was measured at 510 nm against a reagent blank.

#### 2.2.3 GC–MS Analysis

The phenolic compounds in the *E. planum* extract were analyzed by gas chromatography–mass spectrometry (GC–MS). Because phenolic compounds are nonvolatile and thermally unstable in their native form, a derivatization step was required to convert them into volatile trimethylsilyl (TMS) derivatives suitable for GC separation. Approximately 5 mg of the dried extract was weighed into a glass vial. For derivatization, 100 μL of pyridine containing 1 mg mL^−1^ of 4‐dimethylaminopyridine (DMAP, catalyst) and 100 μL of N,O‐Bis (trimethylsilyl) trifluoroacetamide (BSTFA) containing 10% trimethylchlorosilane (TMCS) were added. The mixture was vortexed thoroughly and heated at 70°C for 2 h in a dry bath. After cooling to room temperature, the reaction mixture was diluted with 200 μL of ethyl acetate, centrifuged (5000 rpm, 5 min), and the clear supernatant was transferred to a GC vial. Chromatographic separation was performed on an Agilent 7890A gas chromatograph coupled to an Agilent 5975C mass‐selective detector (MSD). A capillary HP‐5MS column (30 m × 0.25 mm i.d., 0.25 μm film thickness) was used. Highpurity helium (99.999%) served as carrier gas at a constant flow of 1.0 mL min^−1^. The injector temperature was set to 280°C and 1 μL of sample was injected in split mode (split ratio 10:1). The oven temperature program was as follows: 60°C (hold 2 min), raised to 180°C at 10°C min^−1^, then to 280°C at 4°C min^−1^, and finally to 300°C at 10°C min^−1^ (hold 5 min). Total run time was 45.5 min. Mass spectra were acquired in electron‐impact (EI) mode at 70 eV; the ion‐source and quadrupole temperatures were maintained at 230°C and 150°C, respectively. The mass range scanned was m/z 50–600. Identification of the TMS‐derivatized compounds was based on matching the acquired mass spectra with the commercial NIST17 and Wiley9n mass‐spectral libraries (match probability > 90%), calculating the Kovats Retention Index (RI) for each peak relative to a C_7_–C_40_ n‐alkane series injected under the same conditions and comparing the obtained RI values with literature RI data reported for phenolic TMS derivatives on similar stationary phases, and co‐injection with authentic reference standards for the most abundant compounds (rosmarinic acid, caffeic acid, quercetin) and other available key phenolics, which confirmed both retention times and mass‐spectral fragmentation patterns. This multi‐criteria approach, leveraging both mass spectral and retention index data, ensures high‐confidence identification of phenolic compounds in the complex plant matrix. Therefore, all phenolic and flavonoid compounds reported in Table 2 are presented as their TMS derivatives, and the listed retention times correspond to these derivatized forms (Ibragic et al. 2021).

#### 2.2.4 Determination of the Antioxidant Activity of the Extract

##### 2.2.4.1 DPPH Free Radical Scavenging Assay

The 2,2‐diphenyl‐1‐picrylhydrazyl (DPPH) free radical scavenging assay was employed to measure the antioxidant activity of various concentrations of the extract (200, 500, 1000, and 2000 ppm), and results were compared with the antioxidant activity of the synthetic antioxidant TBHQ (100 ppm). For this purpose, 0.1 mL of the extract was mixed with 0.9 mL of a 0.15 mM DPPH solution in 95% ethanol. After incubation at room temperature for 30 min, the absorbance of the mixture was measured at a wavelength of 517 nm using a UV–Vis spectrophotometer (Chen, Liang, and Han 2020).

##### 2.2.4.2 β‐Carotene–Linoleic Acid Bleaching Assay

The β‐carotene–linoleic acid bleaching assay was performed according to Salami et al. (2020). Briefly, 5 mg of β‐carotene was dissolved in 10 mL of chloroform to create a stock solution. Then, 600 μL of this solution was mixed with 40 mg of linoleic acid and 400 mg of Tween 40. The chloroform was completely removed under a stream of nitrogen or by rotary evaporation. Subsequently, 100 mL of oxygenated distilled water was added to the mixture and homogenized vigorously to form a stable emulsion. Aliquots (5 mL) of this final emulsion were transferred to test tubes, and 200 μL of the plant extract at various concentrations was added to each tube. The absorbance was measured at 470 nm immediately (*A*_0_) and after incubation at 50°C for 120 min (*A*_120_). The antioxidant activity, expressed as the percentage inhibition of β‐carotene bleaching, was calculated by comparing the degradation rates of the samples with that of a control containing no antioxidant. The results were compared with the synthetic antioxidant TBHQ at 100 ppm (Salami et al. 2020).

#### 2.2.5 Extraction of Gum

BSG was extracted based on Razavi et al. (2009) with minor modifications. Seeds were soaked in deionized water (1:65 w/v) at 68°C, pH 8, and stirred at 1000 rpm for 20 min. The swollen seeds were passed through a mechanical extractor (JC 700P, Pars Khazar, Iran) to remove the gum layer. Remaining gum was recovered by re‐immersing the seeds in water and repeating extraction. Collected gum was combined, filtered, and dried in an oven (Model 4567, Kimia Pars, Iran) at 50°C. The dried gum was ground, packed in airtight bags, and stored in a cool, dry place (Razavi et al. 2009).

#### 2.2.6 Preparation of Nanoemulsions

Nanoemulsions were prepared as described by Yazdan‐Bakhsh et al. (2020) with slight modifications. BSG served as the wall material and *E. planum* extract as the core. Core‐to‐wall ratios of 1:10 and 1:20 and wall concentrations of 1% and 2% were tested (Table 1). BSG solutions were prepared at the specified concentrations using a magnetic stirrer (D500, Alpha Co., Iran) and stored at 4°C for 24 h to complete hydration. A primary emulsion was first prepared by combining the extract (1 g, 10% w/w), Tween 80 (4 g, 40% w/w), and canola oil (5 g, 50% w/w). This primary emulsion was then emulsified with the BSG coating solution for 5 min at 15,000 rpm and 10°C using a high‐speed homogenizer (T18, IKA, Germany). Particle size was further reduced with a probe sonicator (HD3200, Bandelin, Germany) at 20 kHz for 5 min. To inhibit microbial growth, 0.02% (w/v) sodium azide was added. Final nanoemulsions were freeze‐dried (SUBLIMATOR‐VACO5, Ziribos, Germany) at 0.017 mPa and −57°C for 48 h (Chranioti et al. 2015).

**TABLE 1:** Name of the treatments studied in the research.

Sample name	Wall type	Wall concentration (%)	Core:wall ratio
BSG1 1:10	Basil seed gum	1	1:10
BSG1 1:20	Basil seed gum	1	1:20
BSG2 1:10	Basil seed gum	2	1:10
BSG2 1:20	Basil seed gum	2	1:20

Abbreviations: BSG 1 1:10, nanocapsules containing *Eryngium planum* extract with a wall of basil seed gum, wall concentration 1%, core:wall ratio 1:10; BSG 1 1:20, nanocapsules containing *E. planum* extract with a wall of basil seed gum, wall concentration 1%, core:wall ratio 1:20; BSG 2 1:10, nanocapsules containing *E. planum* extract with a wall of basil seed gum, wall concentration 2%, core:wall ratio 1:10; BSG 2 1:20, Nanocapsules containing *E. planum* extract with a wall of basil seed gum, wall concentration 2%, core:wall ratio 1:20.

#### 2.2.7 Characterization of Nanocapsules and Encapsulation Efficiency

Particle size, zeta potential, and polydispersity index (PDI) were measured using a DLS‐Zeta Sizer (Nano ZS90, Malvern, UK) at 25°C (Ebrahimi et al. 2025). Encapsulation efficiency was calculated following Jafari et al. (2022) using the equation:

$$\text{Encapsulation efficiency}\;\left(\%\right)=\left(\frac{\mathrm{TPC}-\mathrm{SPC}}{\mathrm{TPC}}\right)\times 100$$

In this equation, SPC was the amount of surface phenol and TPC was the amount of total phenol (Jafari et al. 2022).

#### 2.2.8 Release Rate Measurement

Nanocapsules (5 g) were dispersed in 5 g phosphate buffer (pH 7), centrifuged for 90 min at 1500 rpm, and the lower phase analyzed in triplicate (*n* = 3). Total phenolic content was measured by the Folin–Ciocalteu method. The release rate was calculated using the following equation, where *R*2 is the phenolic content of the outer phase and *R*1 that of the inner phase (Kenari et al. 2020):

$$\text{Release rate}\;\left(\%\right)=100-\left(100\times \frac{R2}{R1}\right)$$

#### 2.2.9 Microscopic Structure Analysis

The morphology of the nanocapsules was examined using a scanning electron microscope (SEM; Philips XL 30S FEG). The nanocapsules were mounted onto aluminum stubs using double‐sided adhesive tape and coated with a thin conductive layer of gold to overcome their nonconductive nature. The prepared samples were then placed in the vacuum chamber. An accelerated electron beam with an operating voltage of 30 kV was directed onto the surface of the samples. Images were obtained based on the backscattered electron signals from the sample surface (Kalušević et al. 2017).

#### 2.2.10 Measurement of Oxidation in Canola Oil

Oxidation levels of canola oil samples containing the nanoencapsulated extract (equivalent to 2000 ppm extract), free extract (2000 ppm), TBHQ (100 ppm), and a control were measured using peroxide value (PV) and p‐anisidine value (PAnV) tests. Samples were incubated at 60°C for 24 days and analyzed on days 0, 4, 8, 12, 16, 20, and 24 (Safarpour et al. 2023).

##### 2.2.10.1 Peroxide Value

The analysis of PV followed the official method Cd 8‐53 from AOCS. First, 30 mL of acetic acid and chloroform solution in a ratio of 3:2 was added to 5 g of the sample and mixed well until it was dissolved. Next, 0.5 mL of saturated potassium iodide solution was added, and the mixture was placed in the dark for 1 min. Subsequently, 30 mL of distilled water was incorporated into the reaction mixture. The solution was titrated with 0.01 N thiosulfate until the yellow color disappeared. During the titration, the mixture was stirred continuously to ensure that iodine was separated from the chloroform layer. Afterwards, 0.5 mL of starch indicator was added. Sodium thiosulfate (0.01 N) was used to titrate the solution until the blue coloration faded, signifying the end of the reaction. Finally, the PV was calculated in terms of meq O_2_/kg of sample and through the following equation:

$$\mathrm{PV}\;\left(\mathrm{meq}\;O_{2}/\mathrm{kg}\right)=\frac{\left(V_{2}-V_{1}\right)\times N\times 1000}{m}$$

In this equation, *V*_1_ and *V*_2_ refer to the volumes of sodium thiosulfate consumed for the blank and test samples, respectively; *m* denotes the sample weight (g), and *N* represents thiosulfate normality (AOCS 2009).

##### 2.2.10.2 p‐Anisidine Value

The PAnV of the oil samples was determined using the method described by Ye et al. (2015). Initially, oil samples (2 mL) were blended with 23 mL of isooctane. The absorbance of this solution (Ab) was measured at a wavelength of 350 nm using a spectrophotometer. Following this, 1 mL of p‐anisidine solution was added to 5 mL of the diluted sample, incubated for 10 min, and the absorbance (As) was measured at 350 nm. The PAnV was calculated using the following equation:

$$\text{PAnV}=25\times \left(1.2\;\mathrm{As}-\mathrm{Ab}\right)/m$$

The variables As and Ab represent the absorbance of the reaction mixture and the oil sample solution, respectively, while *m* is the mass of oil analyzed (Ye et al. 2015).

### 2.3 Statistical Analysis

Experimental data were analyzed with IBM SPSS Statistics (Version 27.0.1.0; IBM Corp., Armonk, NY, USA) using ANOVA in a completely randomized design. Mean comparisons were performed with Duncan's test at a 95% confidence level (*p* < 0.05). All experiments were performed in technical triplicate (*n* = 3) using a single batch of *E. planum* extract. Therefore, statistical inferences are valid for comparisons within this experimental setup. Graphs were created using Microsoft Excel (Version 2021; Microsoft Corp., Redmond, WA, USA).

## 3 Results and Discussion

### 3.1 Total Phenolic and Flavonoid Contents in *Eryngium planum* Extract

Phenolic and flavonoid compounds represent key secondary metabolites derived from the shikimate‐phenylpropanoid biosynthetic pathway. The antioxidant capacity of these compounds primarily stems from hydroxyl groups within their molecular architecture, enabling effective neutralization of free radicals and contribution to plant defense systems (Vuolo et al. 2019).

In the present investigation, the *E. planum* extract (EPE) demonstrated a total phenolic content (TPC) of 90.24 mg GAE/g and total flavonoid content (TFC) of 36.21 mg QE/g. These measurements align with the wide range of phenolic and flavonoid levels reported for various *Eryngium* species, highlighting the inherent variability in plant‐based materials.

A comparative assessment of existing literature reveals substantial variation in phenolic and flavonoid contents across different *Eryngium* studies. Kikowska et al. (2022) conducted a comprehensive analysis of three *Eryngium* species ( *E. campestre* , *E. maritimum* , and *E. planum* ), quantifying TPC in leaf extracts at 9.45 ± 0.04, 11.20 ± 0.17, and 12.21 ± 0.12 mg GAE/g, respectively. Their investigation further emphasized substantial variation between plant organs, with root extracts exhibiting TPC of 10.30 ± 0.20, 1.50 ± 0.01, and 2.45 ± 0.01 mg GAE/g for the respective species (Kikowska et al. 2022). Güneş et al. (2014) determined TPC values of 116.69 and 109.62 mg GAE/g in flower and leaf extracts of *Eryngium*, respectively, while Azizkhani and Sodanlo (2021) described a TPC of 120.5 ± 2.4 mg GAE/g (Güneş et al. 2014). Matejić et al. (2018) established TPC and TFC values of 111.9 ± 0.11 mg GAE/g and 164.5 ± 0.05 mg QE/g, respectively, in their analysis of *Eryngium* extract (Matejić et al. 2018).

The observed variability in phenolic and flavonoid contents reflects the complex interplay of multiple analytical and biological factors. Extraction parameters—particularly solvent selection and polarity—interact significantly with genotypic variations, plant organ specificity, developmental stages, and environmental conditions including geographical origin, light exposure, temperature regimes, soil characteristics, and biotic stressors to modulate the production of these compounds (Cirak and Radusiene 2019).

### 3.2 Characterization of Chemical Compounds

Comprehensive phytochemical profiling of *E. planum* extract led to the identification of 21 specific phenolic and flavonoid compounds through GC–MS analysis (Table 2). The analysis was performed on trimethylsilyl derivatives prepared using BSTFA/TMCS, providing reliable identification through dual validation (mass spectra and retention indices) and confirmation with standards for key compounds. Rosmarinic acid (645.79 mg/kg), caffeic acid (413.35 mg/kg), and quercetin (244.76 mg/kg) emerged as the most abundant constituents, aligning with the characteristic phytochemical profile documented for *Eryngium* species and providing a chemical basis for its potent antioxidant activity. This profile shows both consistencies and variations when compared with other studies. Kikowska et al. (2022) identified several phenolic acids—including chlorogenic, isochlorogenic, ferulic, 3,4‐dihydroxyphenylacetic, caffeic, protocatechuic, rosmarinic, syringic, vanillic, and 4‐feruloylquinic acids—along with three flavonoids (kaempferol, quercitrin, and astragalin) in *E. planum* . In their study, rosmarinic acid and chlorogenic acid were reported as the most abundant compounds, which is partially consistent with our findings where rosmarinic acid was also the predominant compound (Kikowska et al. 2022).

**TABLE 2:** Phenolic and flavonoid compounds identified in *Eryngium planum* extract by GC–MS analysis.

Compound amount	Amount (mg/kg, dry weight basis)	Retention time (min)
Astragalin	115.63	2.94
Gallic acid	124.72	3.37
Catechol	136.58	6.52
Protocatechuic acid	2.08	7.45
p‐Hydroxybenzoic acid	96.37	8.13
2‐Methoxy‐4‐vinylphenol	1.05	8.47
Catechin	22.61	9.04
Epicatechin	4.43	9.51
Chlorogenic acid	106.20	10.15
Vanillic acid	192.81	10.26
**Caffeic acid**	**413.35**	**10.45**
Syringic acid	57.69	11.48
p‐Coumaric acid	28.49	12.32
Rutin	39.26	15.25
Ferulic acid	1.14	16.21
Ellagic acid	13.27	17.28
**Rosmarinic acid**	**645.79**	**18.33**
Rosmarinic acid hexoside	108.25	19.54
**Quercetin**	**244.76**	**21.23**
Kaempferol	15.48	25.43
Apigenin	31.15	28.12

*Note:* The three most abundant compounds are highlighted in bold for emphasis.

However, other investigations reveal different dominant compounds. Matiusha et al. (2025) reported chlorogenic acid, sinapic acid, and trans‐cinnamic acid as the predominant compounds in *E. planum* extract, while Paun et al. (2019) identified rutin and isoquercitrin as the primary flavonoids (Matiusha et al. 2025; Paun et al. 2019). The variations in the reported phytochemical profiles of *E. planum* across different studies can be systematically attributed to a complex interplay of intrinsic and extrinsic factors. Intrinsic genetic variations between plant populations or accessions establish the foundational potential for the production of specific metabolites. These genetic factors interact significantly with extrinsic environmental conditions, including soil composition, sunlight exposure, water availability, and the plant's maturity stage at harvest, all of which dynamically modulate biosynthetic pathways. Furthermore, postharvest methodological choices, particularly the extraction protocol (e.g., solvent type, temperature), act as a decisive filter that selectively recovers certain compound classes, thereby shaping the final observed profile (Mohammadi Bazargani et al. 2021; Sabindo et al. 2024). Therefore, the profile rich in rosmarinic acid reported herein is the result of the specific confluence of genetic, environmental, and methodological factors for our plant source and protocol. Crucially, despite this inherent variability, the consistent identification of potent antioxidant phenolics like rosmarinic acid across multiple studies confirms the robust potential of *E. planum* extract for applications in oxidative stabilization, which is the central focus of this research.

### 3.3 Antioxidant Activity

#### 3.3.1 DPPH Free Radical Scavenging Activity

**FIGURE 1:**
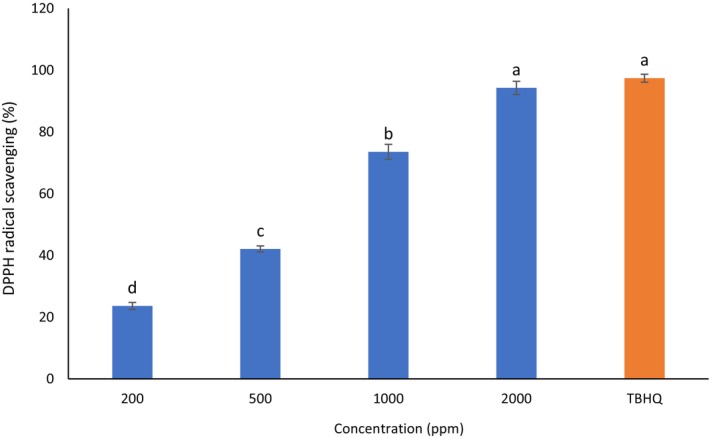
DPPH radical scavenging activity by different concentrations of *Eryngium planum* extract and TBHQ (100 ppm). Data are presented as mean ± standard deviation of triplicate measurements from a single extract batch. Different letters indicate significant differences (*p* < 0.05). TBHQ, tert‐butyl hydroquinone.

The radical scavenging capacity of the EPE was evaluated by the DPPH assay, which measures the reduction of the stable DPPH radical following hydrogen donation from antioxidants (Gulcin and Alwasel 2023). As depicted in Figure 1, EPE exhibited a pronounced dose‐dependent scavenging activity, with inhibition rates rising significantly as concentration increased (*p* < 0.05). Notably, the activity at 2000 ppm was statistically on par with that of 100 ppm TBHQ, demonstrating a potent capacity to quench DPPH radicals comparable to the synthetic standard. This strong concentration–response relationship is consistent with the high phenolic content of the extract, wherein greater concentrations supply more hydrogen‐donating hydroxyl groups to neutralize free radicals (Vuolo et al. 2019).

Consistent with our findings, Daneshzadeh et al. (2020) reported a DPPH inhibition rate of 51.63% for *Eryngium billardieri* extract at 500 ppm. The observed variations in activity between *E. billardieri* and *E. planum* at comparable intermediate concentrations can be attributed to inherent differences in their specific bioactive compound profiles, which are influenced by factors such as genetic background and environmental conditions (Tripodi et al. 2018). While *E. billardieri* demonstrates notable activity at lower concentrations, a key finding of the present work is that *E. planum* extract achieves a remarkably high efficacy at its peak concentration, matching the performance of the synthetic antioxidant TBHQ. This underscores the significant potential of *E. planum* as a rich source of natural antioxidants capable of reaching potency levels comparable to established synthetic standards.

Our finding of a concentration‐dependent increase in antioxidant activity is a common trend observed in various plant extracts. For instance, Rezaei Savadkouhi et al. (2020) found a direct correlation between phenolic content and DPPH scavenging activity in *Hyssopus officinalis* L. extract. In line with this, Aziminezhad et al. (2024) also reported a dose‐responsive antioxidant response in their evaluation of plant extracts. Furthermore, Bordbar Lomer and Ghannadiasl (2025) reported a concentration‐dependent increase in antioxidant potential for cinnamon extract, attributing this effect to the greater density of bioactive compounds at elevated levels. Other researchers, including Delfanian et al. (2016) and Kenari et al. (2020), have likewise reported parallel findings, further supporting the results of the current study.

#### 3.3.2 β‐Carotene–Linoleic Acid Bleaching Activity

**FIGURE 2:**
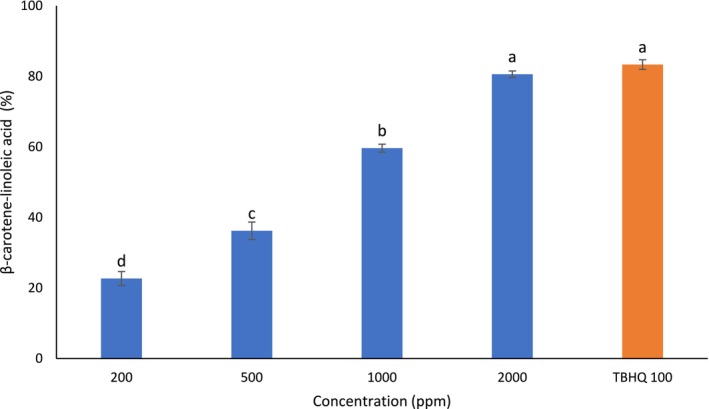
Bleaching of β‐carotene–linoleic acid of different concentrations of *Eryngium planum* extract and TBHQ (100 ppm). Data are presented as mean ± standard deviation of triplicate measurements from a single extract batch. Different letters indicate significant differences (*p* < 0.05). TBHQ, tert‐butyl hydroquinone.

To assess the antioxidant capacity of EPE, the β‐carotene–linoleic acid model system was used to monitor its effectiveness against lipid‐derived radicals. In this reaction system, lipid‐derived radicals degrade β‐carotene molecules, leading to a noticeable decrease in the orange coloration of the solution, which serves as an indicator of oxidative activity (Ahmed et al. 2022). As shown in Figure 2, a significant enhancement in the extract's antioxidant capacity was observed with increasing concentration, ranging from 200 to 2000 ppm (*p* < 0.05). This indicates a concentration‐dependent protective effect against β‐carotene degradation, attributable to antioxidant compounds in the reaction matrix (Alam et al. 2013). This concentration‐dependent efficacy aligns with observations for other plant extracts in the β‐carotene–linoleic acid system (Jafari et al. 2022; Souad et al. 2025). Importantly, at a concentration of 2000 ppm, the antioxidant performance of EPE did not differ significantly from that of the synthetic antioxidant TBHQ (*p* > 0.05), suggesting a comparable efficacy in mitigating lipid peroxidation. This result concurs with findings for other potent plant extracts, such as loquat fruit, which also achieved performance similar to TBHQ in this model system (Delfanian et al. 2015b).

Collectively, these findings highlight the strong antioxidant capability of EPE and its potential as a natural alternative to synthetic antioxidants. Based on the superior antioxidant performance at 2000 ppm, this concentration was selected for subsequent encapsulation processes.

### 3.4 Characterization of Nanocapsules

The physicochemical properties of nanoemulsions, particularly particle size, are critical determinants of their stability and functionality (Ilangovan et al. 2021; McClements et al. 2009). As illustrated in Table 3, all nanocapsules exhibited nanometric particle sizes, with the smallest size observed for the BSG2 1:20 nanocapsule (59.8 ± 0.79 nm). A significant reduction in particle size was observed with increasing wall material concentration, potentially attributable to enhanced emulsion stability and suppressed droplet coalescence through increased continuous phase viscosity (Kupikowska‐Stobba et al. 2024; McClements 2004). Furthermore, the core‐to‐wall ratio had a statistically significant influence on particle size, with lower ratios correlating with reduced particle dimensions (*p* < 0.05). This trend aligns with the observations of Frascareli et al. (2012), where increasing gum concentration resulted in smaller particle sizes. Mahalakshmi et al. (2023) encapsulated curcumin using whey protein through two drying methods: spray drying and electrospraying, at two core‐to‐wall ratios of 1:200 and 1:500. They reported that the particle size was smaller at the 1:500 core‐to‐wall ratio compared to the 1:200 ratio in both encapsulation techniques (Mahalakshmi et al. 2023).

**TABLE 3:** Nanocapsules characteristics.

Sample name	Particle size (nm)	polydispersity index (PDI)	Zeta potential (mV)	Encapsulation efficiency (%)
BSG1 1:10	74.6 ± 0.49^a^	0.246 ± 0.004^a^	−20.94 ± 0.84^c^	68.03 ± 0.29^d^
BSG1 1:20	64.9 ± 0.36^b^	0.235 ± 0.007^ab^	−22.01 ± 0.51^b^	79.45 ± 0.16^c^
BSG2 1:10	63.1 ± 0.16^c^	0.217 ± 0.010^bc^	−34.45 ± 0.15^a^	80.62 ± 0.49^b^
BSG2 1:20	59.8 ± 0.79^d^	0.204 ± 0.005^c^	−36 ± 0.57^a^	86.95 ± 0.33^a^

*Note:* Data are presented as mean ± standard deviation of triplicate measurements from a single extract batch. Different lowercase letters in the same column indicate significant differences (*p* < 0.05).

Abbreviations: BSG, basil seed gum; BSG 1 1:10, nanocapsules containing *Eryngium planum* extract with a wall of basil seed gum, wall concentration 1%, core:wall ratio 1:10; BSG 1 1:20, nanocapsules containing *E. planum* extract with a wall of basil seed gum, wall concentration 1%, core:wall ratio 1:20; BSG 2 1:10, nanocapsules containing *E. planum* extract with a wall of basil seed gum, wall concentration 2%, core:wall ratio 1:10; BSG 2 1:20, nanocapsules containing *E. planum* extract with a wall of basil seed gum, wall concentration 2%, core:wall ratio 1:20.

The polydispersity index (PDI), which quantifies the uniformity of particle size distribution, typically ranges between 0 and 1 (Raval et al. 2019). As reported in Table 3, all nanoemulsions demonstrated PDI values below 0.3, suggesting a homogeneous distribution of particle sizes and indicating good kinetic stability of the colloidal system (Aboutalebzadeh et al. 2022). Although core‐to‐wall ratio had no significant effect on PDI, increasing wall concentration contributed to a more uniform particle size distribution. Smaller particle sizes tend to be associated with narrower size distributions. The extent of particle disruption during emulsification, governed by homogenization pressure and processing intensity, also plays a pivotal role in achieving low PDI values (Yuan et al. 2008). The current study employed high‐pressure homogenization combined with ultrasonication, resulting in well‐dispersed, uniformly sized nanoparticles with high stability.

Zeta potential, an indicator of surface charge in colloidal systems, is a crucial parameter for assessing electrostatic stability (Astutiningsih et al. 2022). Values exceeding ±30 mV typically signify strong repulsive forces and greater colloidal stability, preventing particle aggregation and coalescence during storage (Karmakar 2019; Kenari and Razavi 2022; Razavi and Kenari 2021). Zeta potential is subject to variation based on several influencing factors such as the ionic strength of the medium, wall material properties, core‐to‐wall ratio, and environmental pH (Kupikowska‐Stobba et al. 2024). As detailed in Table 3, nanoemulsions BSG2 1:20 and BSG2 1:10 showed zeta potential values below −30 mV, indicative of high physical stability owing to substantial electrostatic repulsion between particles (Lu and Gao 2010). Increased wall concentration led to significantly higher absolute zeta potential values, while core‐to‐wall ratio at 2% wall concentration did not cause marked changes. The overall negative surface charge observed across all samples is attributable to the anionic nature of the gums. The study by Jafari et al. (2022) also reported a zeta potential of −24.35 mV for a nanoemulsion prepared with basil seed gum. Other researchers also reported negative zeta potential for *H. officinalis* nanoencapsulated extract in basil seed gum (Rezaei Savadkouhi et al. 2020), *Fumaria parviflora* extract encapsulated in gum arabic (Razavi and Kenari 2021), and sesame seed phenolic compounds in native seed gums (Esmaeilzadeh Kenari and Razavi 2022), indicating the anionic nature of the gums.

Encapsulation efficiency (EE) is another critical metric reflecting the protective capacity of the wall matrix and its ability to prevent leakage of core materials during processing. It indicates the proportion of active compounds successfully retained within the encapsulated structure (Anand et al. 2024). In the present study, EE values ranged from 68.03% ± 0.29% to 86.95% ± 0.33%, with the highest efficiency observed in BSG2 1:20 nanocapsules. The superior encapsulation performance can be directly attributed to the distinctive structural and physicochemical properties of basil seed gum (BSG). BSG contains high molecular weight polysaccharides with exceptional matrix‐forming capacity, which during the freeze‐drying process facilitates the development of a continuous and robust wall structure around the extract‐containing oil droplets, thereby effectively preventing core material leakage (Guan et al. 2023; Pirsa and Hafezi 2023).

A higher wall‐materials concentration positively affected EE, and increasing the wall ratio from 1:10 to 1:20 produced a clear improvement. Various parameters such as wall type and concentration, core properties, core‐to‐wall ratio, particle size, viscosity, drying method, and emulsion characteristics can significantly impact EE outcomes (Sadati Khadar et al. 2024). In general, the increased EE at higher BSG concentrations (2%) aligns with the enhanced viscosity and gel‐forming ability of concentrated BSG solutions, which reduce droplet mobility and coalescence, thereby minimizing core material leakage (Guan et al. 2023).

Our findings are corroborated by previous research. Ding et al. (2020) demonstrated that total solids content, core‐to‐wall material ratio, and wall material type significantly impact encapsulation efficiency. They noted that insufficient wall material prevents the formation of a dense, continuous shell, thereby reducing efficiency, while increasing total solids enhances it (Ding et al. 2020). Similarly, Sekhavatizadeh et al. (2025) attributed reduced EE to inadequate wall material for robust matrix formation, and improved efficiency to rapid shell development on particle surfaces. Zhou et al. (2017) emphasized that the most influential factor for encapsulation performance was the wall material concentration.

Various studies have shown that particle size can also have a significant impact on encapsulation efficiency; larger particles generally show lower EE because delayed shell formation allows more core leakage during drying (Aboutalebzadeh et al. 2022; Frascareli et al. 2012). Consistent with these reports, our study revealed a clear inverse relationship between particle size and encapsulation efficiency, with smaller nanocapsules such as BSG2 1:20 demonstrating the highest EE values. This trend is also validated by Teng et al. (2012), who reported a similar inverse relationship. Azizkhani and Sodanlo (2021) also reported in a study that there is a significant correlation between the decrease in droplet diameter and the increase in EE of *Eryngium campestre* extract, and smaller droplets showed higher EE.

### 3.5 Release Kinetics

The release profile of phenolic compounds from nanocapsules during storage was evaluated, as their controlled release directly influences antioxidant efficacy and product shelf life (Roshanpour et al. 2021). As shown in Table 4, all nanocapsules exhibited low initial release (approximately 2%–5% on day 0), confirming successful encapsulation. This effective initial protection can be attributed to the rapid formation of a dense physical barrier by the BSG matrix at the particle interface.

**TABLE 4:** Release rate (%) of phenolic compounds from nanocapsules.

Sample name	Storage time (day)						
	0	4	8	12	16	20	24
BSG 1 1:10	2.85 ± 0.08^Ag^	14.22 ± 0.27^Af^	25.18 ± 0.76^Ae^	39.89 ± 0.64^Ad^	54.36 ± 0.40^Ac^	67.62 ± 0.63^Ab^	83.52 ± 0.39^Aa^
BSG 1 1:20	2.57 ± 0.05^Ag^	14.35 ± 0.14^Af^	23.57 ± 0.18^Be^	37.64 ± 0.19^Bd^	52.41 ± 0.35^Bc^	65.81 ± 0.89^ABb^	80.19 ± 0.24^Ba^
BSG 2 1:10	1.99 ± 0.7^Ag^	13.86 ± 0.82^Af^	21.75 ± 0.14^Ce^	36.93 ± 0.42^Bd^	50.11 ± 0.26^Cc^	64.33 ± 1.29^BCb^	77.29 ± 0.58^Ca^
BSG 2 1:20	1.89 ± 0.55^Ag^	12.39 ± 0.13^Bf^	20.29 ± 0.44^De^	37.26 ± 0.86^Bd^	47.39 ± 0.45^Dc^	62.82 ± 0.51^Cb^	77.2 ± 0.32^Ca^

*Note:* Data are presented as mean ± standard deviation of triplicate measurements from a single extract batch. Different uppercase letters within the same column indicate statistically significant differences, whereas different lowercase letters within the same row indicate significant differences at *p* < 0.05.

Abbreviations: BSG, basil seed gum; BSG 1 1:10, nanocapsules containing *Eryngium planum* extract with a wall of basil seed gum, wall concentration 1%, core:wall ratio 1:10; BSG 1 1:20, nanocapsules containing *E. planum* extract with a wall of basil seed gum, wall concentration 1%, core:wall ratio 1:20; BSG 2 1:10, nanocapsules containing *E. planum* extract with a wall of basil seed gum, wall concentration 2%, core:wall ratio 1:10; BSG 2 1:20, nanocapsules containing *E. planum* extract with a wall of basil seed gum, wall concentration 2%, core:wall ratio 1:20.

Subsequently, a significant time‐dependent increase in phenolic release was observed across all formulations (*p* < 0.05). This controlled‐release profile is governed by the structural integrity and gel‐forming capacity of the BSG matrix. The gradual swelling and hydration of the polysaccharide network in the aqueous environment create a diffusion‐controlled system, which is further modulated by the gradual degradation of the wall matrix, enabling the sustained diffusion of the encapsulated core (Czyzynska‐Cichon et al. 2021; Guan et al. 2023; Pirsa and Hafezi 2023).

The release kinetics were significantly influenced by the wall concentration and core‐to‐wall ratio. An increased wall‐to‐core ratio consistently led to a reduced release rate. This is because a higher proportion of wall material forms a denser, more cohesive, and less permeable matrix during drying, creating a longer and more tortuous diffusion path for the phenolic compounds and thereby enhancing their retention (Aboutalebzadeh et al. 2022). Consequently, by the end of the storage period, the highest cumulative release was observed in samples with lower wall ratios, namely BSG1 1:10, BSG1 1:20, BSG2 1:10, and BSG2 1:20, respectively.

In a related study, the release of rosemary extract encapsulated with cress and basil seed gums demonstrated a time‐dependent release pattern across all samples, with significant statistical differences. Notably, samples prepared with basil seed gum had better release performance than cress ( *Lepidium sativum* ) seed gum (Jafari et al. 2022). Other studies have also reported the gradual release of phenolic compounds from *Ferula persica* extract (Estakhr et al. 2020), olive leaf (Mohammadi et al. 2016), and olive pomace (Paini et al. 2015).

### 3.6 Morphological Characteristics of Nanocapsules

**FIGURE 3:**
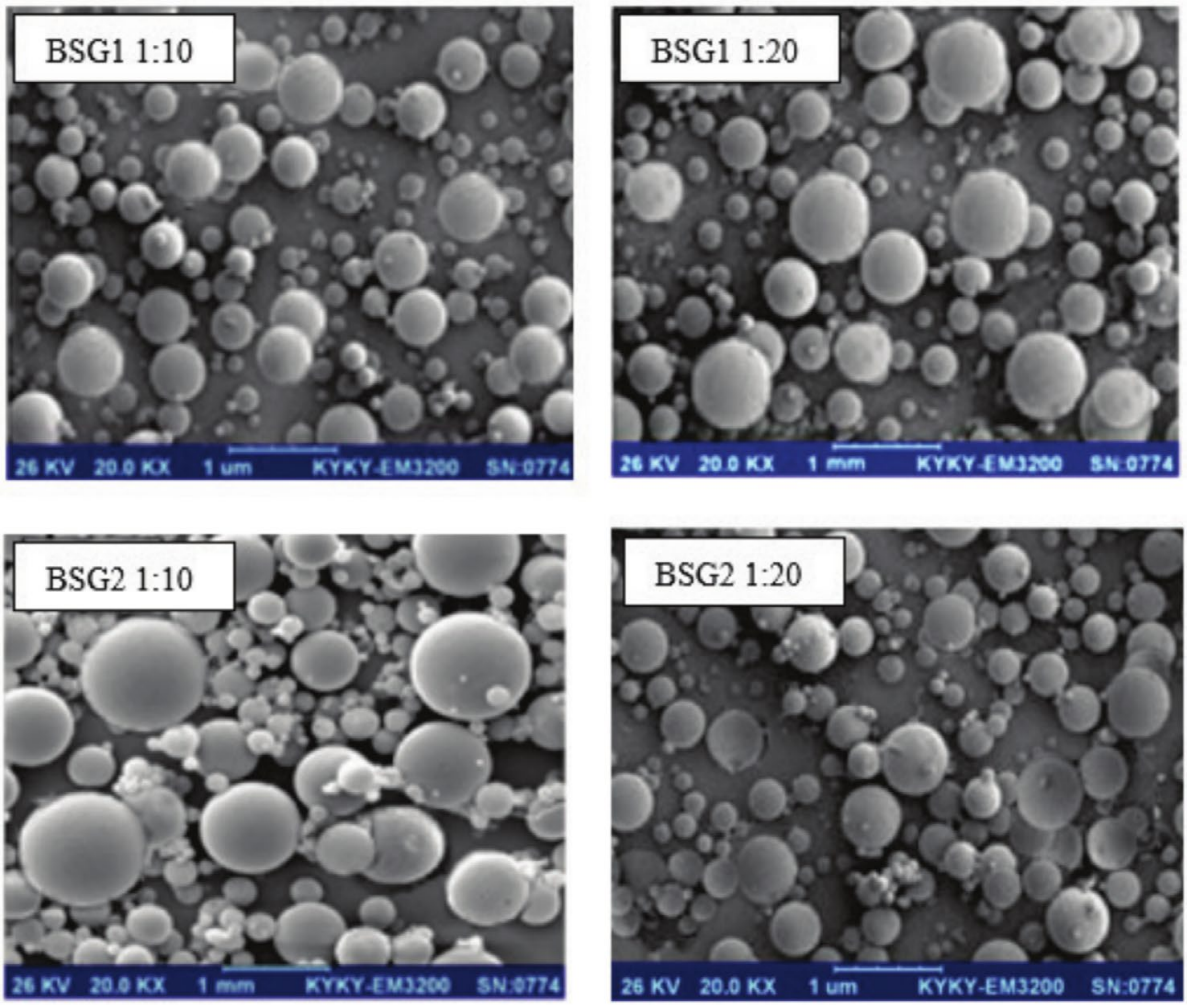
Scanning electron microscope images of nanocapsules. BSG, basil seed gum; BSG 1 1:10, nanocapsules containing *Eryngium planum* extract with a wall of basil seed gum, wall concentration 1%, core:wall ratio 1:10; BSG 1 1:20, nanocapsules containing *E. planum* extract with a wall of basil seed gum, wall concentration 1%, core:wall ratio 1:20; BSG 2 1:10, nanocapsules containing *E. planum* extract with a wall of basil seed gum, wall concentration 2%, core:wall ratio 1:10; BSG 2 1:20, nanocapsules containing *E. planum* extract with a wall of basil seed gum, wall concentration 2%, core:wall ratio 1:20.

Surface morphology plays a pivotal role in determining the encapsulation efficiency of plant‐based extracts (Kenari et al. 2020). In this study, scanning electron microscopy (SEM) was employed to assess the structural features of BSG‐based nanocapsules. As depicted in Figure 3, the particles displayed spherical shapes with smooth, wrinkle‐free surfaces and no observable pores, confirming effective moisture removal during drying and successful encapsulation. Particles exhibiting more uniform and spherical morphology tend to provide better protection against environmental moisture and oxygen, thereby enhancing the long‐term stability of bioactives. Studies have shown that wall material selection, drying rate, and process parameters considerably influence surface properties (Aziminezhad et al. 2024; Kenari et al. 2020). Incomplete moisture removal can lead to irregular, collapsed particles with rough textures, a phenomenon not observed in the current samples (Nijdam and Langrish 2006; Tonon et al. 2008).

Our SEM observations are consistent with those of Sayyari et al. (2021), who reported spherical structures in nanocapsules with walls composed of *L. perfoliatum* , basil, and their complexes. Comparable morphologies have also been documented for capsules containing *Salvia officinalis* L. (Safarpour et al. 2023), *H. Lasiopetalum* (Yazdan‐Bakhsh et al. 2020), *Pistacia khinjuk* (Hosseinialhashemi et al. 2021), and sesame seed extracts (Esmaeilzadeh Kenari and Razavi 2022).

### 3.7 Oxidative Stability of Canola Oil

#### 3.7.1 Peroxide Value

**FIGURE 4:**
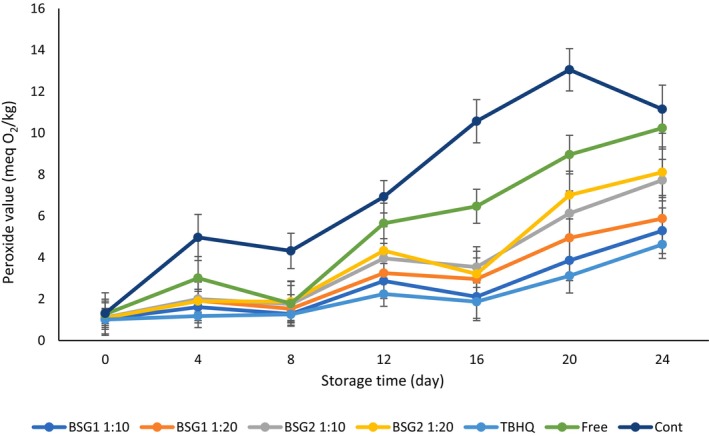
Changes in peroxide value of canola oil during storage. Data are presented as mean ± standard deviation of triplicate measurements from a single extract batch. BSG, basil seed gum; BSG 1 1:10, nanocapsules containing *Eryngium planum* extract with a wall of basil seed gum, wall concentration 1%, core:wall ratio 1:10; BSG 1 1:20, nanocapsules containing *E. planum* extract with a wall of basil seed gum, wall concentration 1%, core:wall ratio 1:20; BSG 2 1:10, nanocapsules containing *E. planum* extract with a wall of basil seed gum, wall concentration 2%, core:wall ratio 1:10; BSG 2 1:20, nanocapsules containing *E. planum* extract with a wall of basil seed gum, wall concentration 2%, core:wall ratio 1:20; Cont, control; Free, free extract; TBHQ, tert‐butyl hydroquinone.

PV is a critical marker for primary lipid oxidation, representing the accumulation of hydroperoxides during the early stages of oxidative degradation (Delfanian et al. 2015a; Hori et al. 2019). Elevated PV levels indicate increased lipid peroxidation. Excessive PV levels in edible oils can result in sensory degradation and potential health hazards, underscoring the importance of regular PV monitoring (Zhang et al. 2021). As shown in Figure 4, initial PV levels were low but increased significantly during storage (*p* < 0.05). The control sample showed the highest PV, likely due to the absence of antioxidant agents. Comparatively, the free extract exhibited weaker antioxidant activity than its nanoencapsulated form. The superior performance of nanoencapsulated extracts over free extract, despite minimal initial phenolic release observed in Section 3.5, provides compelling evidence for the protective function of the nanocapsules. While the free extract may show initial antioxidant activity, it rapidly degrades when directly exposed to the oxidative environment of the oil. In contrast, the nanocapsules maintain a sustained release of fresh antioxidants throughout storage, explaining their significantly better long‐term performance (Lankanayaka et al. 2025; Wangsuntornpakdee et al. 2025). By day 24, the lowest PV among nanocapsulated samples was recorded in BSG1 1:10, with no significant difference compared to BSG1 1:20 (*p* > 0.05). Both formulations showed antioxidant activity approaching that of the synthetic antioxidant TBHQ, although TBHQ still exhibited the lowest absolute PV values.

Consistent with our findings, Delfanian et al. (2018) produced nanoemulsions of Bene hull polyphenols ( *Pistacia atlantica* subsp. *mutica*) using soy protein isolate and basil seed gum (SPI‐BSG) and whey protein isolate and basil gum (WPI‐BSG) and investigated their performance in soybean oil. The results indicated that the encapsulated forms consistently outperformed the free extract, with SPI‐BSG showing superior antioxidant efficacy due to controlled phenolic release. Nevertheless, TBHQ maintained the highest performance overall (Delfanian et al. 2018). Roshanpour et al. (2021) reported that the highest peroxide value at the end of a 24‐day storage period at 60°C was observed first in pure soybean oil and then in the sample containing TBHQ, while the lowest peroxide value was obtained by nanoencapsulating the phenolic extract of *M. piperita*. Tavakoli et al. (2023) also stated that nanoencapsulation had a positive effect on the antioxidant activity of *Mentha aquatica* extract, with the nanoencapsulated extract (200 ppm) showing higher antioxidant activity than TBHQ. In another study, the antioxidant activity of free and nanoencapsulated *E. campestre* extract in chitosan (CS) and maltodextrin (MD) was evaluated in canola oil under accelerated oxidation conditions. The nanoencapsulation process increased the antioxidant power of the extracts, and the formulation containing 8.5% MD + 1.5% CS showed the best antioxidant effect in canola oil (Azizkhani and Sodanlo 2021). Additionally, Aghakeshipour et al. (2025) noted the high antioxidant efficacy of nanoencapsulated corn husk extract in basil seed gum and sesame protein isolate (Aghakeshipour et al. 2025). These results indicate the positive role of plant compounds in increasing the oxidative stability of the oil and the higher effectiveness of the nanoencapsulated form in preserving these compounds against oxidative degradation. Similar findings have also been reported, all of which emphasized the increased effectiveness of natural antioxidants in nanoencapsulated form in reducing the oxidation of edible oils (Bagheri et al. 2016; Faria et al. 2020; Taghvaei et al. 2014).

#### 3.7.2 p‐Anisidine Value

PAnV is a reliable indicator of secondary oxidation products, including aldehydes, ketones, and carbonyl compounds such as 2‐alkenals and 2,4‐alkadienals (Faria et al. 2020). These compounds contribute to off‐flavors; thus, oils with lower PAnV are generally of higher sensory quality during early storage (Hosseinialhashemi et al. 2021). PAnV below 10 is typically considered indicative of good oil quality (Khoddami and Roberts 2015).

**FIGURE 5:**
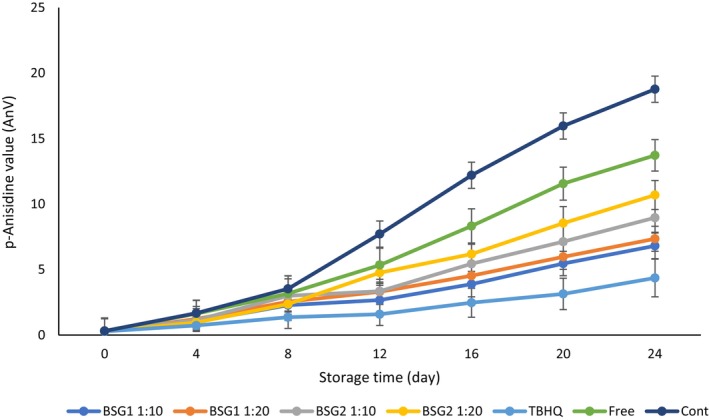
Changes in p‐anisidine value of canola oil during storage. Data are presented as mean ± standard deviation of triplicate measurements from a single extract batch. BSG, basil seed gum; BSG 1 1:10, nanocapsules containing *Eryngium planum* extract with a wall of basil seed gum, wall concentration 1%, core:wall ratio 1:10; BSG 1 1:20, nanocapsules containing *E. planum* extract with a wall of basil seed gum, wall concentration 1%, core:wall ratio 1:20; BSG 2 1:10, nanocapsules containing *E. planum* extract with a wall of basil seed gum, wall concentration 2%, core:wall ratio 1:10; BSG 2 1:20, nanocapsules containing *E. planum* extract with a wall of basil seed gum, wall concentration 2%, core:wall ratio 1:20; Cont, control; Free, free extract; TBHQ, tert‐butyl hydroquinone.

As shown in Figure 5, initial PAnV values were low and statistically similar across treatments but increased significantly over time (*p* < 0.05), with the control sample showing the highest increase. The free extract provided only modest protection, delaying but not preventing the rise in PAnV. In contrast, samples containing nanoencapsulated extract consistently exhibited lower PAnV than those with free extract. This pattern aligns with the peroxide value results and confirms the sustained antioxidant protection offered by the nanocapsules. The BSG matrix provides a controlled release of phenolic compounds, which maintains their bioavailability in the oil phase throughout storage. This sustained presence of active antioxidants suppresses the formation of secondary oxidation products, particularly aldehydes. Consistent with our findings, other researchers also observed improved antioxidant function through nanoencapsulation (Hosseinialhashemi et al. 2021; Moczkowska et al. 2020; Tavakoli et al. 2023).

On day 24, the lowest PAnV among encapsulated samples were observed in BSG1 1:10 and BSG1 1:20. The superior performance of these formulations in controlling secondary oxidation can be attributed to their gradual and sustained phenolic release pattern, which ensures continuous availability of active antioxidants throughout the storage period (Sharma et al. 2019). However, their antioxidant activity remained slightly lower than TBHQ. Yazdan‐Bakhsh et al. (2020) reached similar results. In another study, Abbasi et al. (2024) reported an improvement in the oxidative stability of canola oil as a result of adding nanoencapsulated phenolic extract of *Pimpinella affinis* in chitosan, *Salvia macrosiphon* gum, and a 1:1 complex of the two. They stated that the encapsulated extract at a concentration of 300 ppm even exhibited higher antioxidant activity compared to TBHQ (Abbasi et al. 2024). Beyond retarding oxidation, the incorporation of *E. planum* extract nanocapsules into canola oil presents a potential nutritional advantage. It is well‐established that encapsulation systems can protect oils' endogenous bioactive compounds, such as tocopherols, phytosterols, and unsaturated fatty acids, from oxidative degradation (Mazzocchi et al. 2021; Popovici et al. 2022; Sharma et al. 2019; Yang et al. 2024). Concurrently, in the present study, the nanocapsules serve as a delivery vehicle to fortify the oil with additional natural antioxidants from the extract, notably rosmarinic acid and caffeic acid. This dual functionality—preserving inherent nutraceuticals while supplementing exogenous ones—could significantly enhance the nutraceutical value of the fortified oil, contributing to a cleaner label product.

## 4 Conclusion

The increasing public concern over the adverse effects of synthetic antioxidants has intensified the demand for natural, plant‐derived alternatives. This study demonstrated that the antioxidant capacity of *E. planum* extract significantly increased with higher concentrations, achieving comparable efficacy to TBHQ at 2000 ppm. Among the encapsulation variables, wall material concentration had a greater influence on the physicochemical properties of the nanocapsules than the core‐to‐wall ratio. The encapsulated extract showed superior oxidative stability performance in canola oil compared to the free extract, with the most effective result observed in the BSG1 1:10 sample. These results highlight the potential of basil seed gum‐based nanocapsules as a natural strategy to improve the shelf life and oxidative stability of edible oils.

It is important to note that this study was conducted using a single batch of *E. planum* extract. While this approach provided necessary internal consistency for evaluating the effects of wall concentration and core‐to‐wall ratio, future research should incorporate multiple, independently sourced extract batches to assess the robustness of the encapsulation system against natural variations in the plant's phytochemical profile and to confirm reproducibility for industrial applications. Further investigations into the rheological properties of these nanoemulsions and their application in other food matrices are also recommended to facilitate industrial scale‐up.

## Author Contributions

**Nastaran Fallah:** writing – original draft, formal analysis, methodology, software, visualization. **Reza Esmaeilzadeh Kenari:** conceptualization, investigation, writing – review and editing, project administration, supervision, data curation. **Reza Farahmandfar:** investigation, project administration, supervision, writing – review and editing.

## Conflicts of Interest

The authors declare no conflicts of interest.

## Data Availability

The data that support the findings of this study are available from the corresponding author upon reasonable request.
